# Influence of Quaternary Ammonium Salt Functionalized Chitosan Additive as Sustainable Filler for High-Density Polyethylene Composites

**DOI:** 10.3390/ma15217418

**Published:** 2022-10-22

**Authors:** Maria José G. de Araújo, Francivandi C. Barbosa, Marcus Vinícius L. Fook, Suédina Maria L. Silva, Itamara F. Leite

**Affiliations:** 1 Graduate Program in Science and Materials Engineering, Federal University of Paraíba, João Pessoa 58051-900, Brazil; 2 Department of Materials Engineering, Federal University of Campina Grande, Campina Grande 58429-900, Brazil; 3 Department of Materials Engineering, Federal University of Paraíba, João Pessoa 58051-900, Brazil

**Keywords:** HDPE, chitosan, quaternary ammonium, additive, antimicrobial activity

## Abstract

In this study, an antimicrobial packaging material was successfully developed with blends of high-density polyethylene (HDPE) and chitosan (CS) made by melt processing. In the different HDPE/CS composites, the CS content effect (up to 40%), and the addition of quaternary ammonium salt functionalized chitosan (CS-CTAB) as an additive were evaluated by X-ray diffraction (XRD), differential scanning calorimetry (DSC), thermogravimetric analyses (TG), tensile strength, scanning electron microscopy (SEM) and antimicrobial activity. When analyzing the effect of the additive in the different HDPE/CS composites, it was observed that the compositions with 10 and 20 %wt of chitosan showed better elongation values (~13% and 10%) as well as a higher decomposition temperature at 20% mass loss (T20) varying from (321–332 °C and 302–312 °C), respectively, in relation to the other compositions, regardless of the type of additive used, it acted as an antimicrobial agent, promoting inhibition of microbial growth against the strains gram-positive and gram-negative used in this work, making the different HDPE/CS composites suitable candidates for use in food packaging.

## 1. Introduction

High-density polyethylene (HDPE) from fossil fuels, has aroused much interest due to its fabulous thermal, mechanical, barrier properties, low cost, and ease of production. Thus, it is one of the most consumed polymers in the world in the production of packaging, as well as, everyday utensils such as buckets, bowls, pots, and toys, among others [1]. However, despite such advantages, plastic packaging has a large impact on the environment and human health due to the non-biodegradable nature of the plastics and the presence of additives in the formulations of plastic packaging, aiming to improve their properties, that can migrate to food during processing and storage [2]. Thus, to reduce the impact of petroleum-based plastic packaging and to improve food quality, various alternative solutions are being attempted. Among them, packaging from bio-sourced and biodegradable plastics is an eco-friendly and sustainable solution. Nevertheless, the high cost, land availability to produce raw materials, durability level, and material performance compared to petroleum-based plastics are considered drawbacks to taking complete advantage of biodegradable plastics for socio-economic benefits [3]. Alternatively, polymer blends and composites containing natural polymers such as collagen, starch, elastin, and chitosan as biodegradable additives have been developed [4,5,6,7,8,9,10,11,12]. The production process of these types of systems is relatively easy and inexpensive and can be commercialized [13].

Chitosan is an abundant natural polymer, derived from the deacetylation of chitin (a homopolymer of β(1–4) linked *N*-acetyl-d-glucosamine) obtained from the shells of crustaceans [14,15]. As a natural renewable resource, chitosan has a number of unique properties such as being biocompatible, biodegradable, and non-toxic, and can interact with metal ions, dyes, proteins, nucleic acids, lipids, herbicides, pesticides, and humic acids [16,17,18]. Chitosan is a natural polycationic polysaccharide and has an excellent antibacterial property against various bacteria, viruses, and fungi [19,20,21]. Generally, antibacterial activity is governed by molecuPlease check intended meaning has been retained.lar weight (Mw), degree of deacetylation, temperature, and pH of solution [22,23,24].

The mixture of HDPE with chitosan is an alternative method for obtaining eco-friendly and high-performing packing materials. In addition, if initially, HDPE does not have antimicrobial and antifungal activities for packaging applications [25,26,27,28,29], by combining with chitosan [4,5,12,30,31], its biodegradation and antimicrobial activity can be improved [32]. Therefore, the use of chitosan as an additive sustainable for polymer-based composites is beginning to receive quite some attention. Chitosan acts as a biodegradable additive and, giving antimicrobial properties, it is a renewable polymer, has relative abundance, is easily biodegradable, inexpensive, has low abrasive nature, and is non-toxic [32,33,34].

Despite the benefits that can be achieved with the use of chitosan as a filler in the HDPE-based composite material for food packaging, research in this regard is still limited [31,35,36,37,38,39,40] and, as far as we know, there is no study concerning the additivation of HDPE/chitosan with quaternary ammonium salt (cetyl trimethyl ammonium bromide-CTAB) functionalized chitosan. Thus, our major interest is to explore the potential of the incorporation of quaternary ammonium salt functionalized chitosan (CS-CTAB), developed in our laboratory, on morphological, thermal, mechanical, and antibacterial properties of HDPE/chitosan composites.

In addition, it is reported in the literature that the synthesis of a chitosan-based antimicrobial agent by salt quaternary ammonium grafting retains its properties and exhibits a remarkable improvement in antibacterial properties compared to unmodified chitosan [41,42,43,44,45,46]. It is important to highlight that in 2018 [38], a work carried out by our research group, synthesized in our laboratory, demonstrated a modified clay with chitosan impregnated with CTAB to be used as a compatibilizer in HDPE/chitosan blends and, therefore, the thermal properties and some of the mechanics were improved, making the mixtures of HDPE/chitosan suitable candidates for food packaging.

In light of this, different formulations of HDPE/chitosan were prepared in this work by melt processing, using quaternary ammonium salt functionalized chitosan as an additive in order to elucidate the effect of these on thermal, morphological, mechanical, and antibacterial properties of HDPE/chitosan composites for food packaging.

## 2. Materials and Methods

### 2.1. Materials

High-density polyethylene (HDPE), GM9450F, with a melt flow index of 9.3 g/10 min (190 °C/2.16 kg) (ASTM D 1238), density of 0.952 g/cm^3^ (ASTM D 792), produced by Braskem (São Paulo, Brazil) was donated by Rava Embalagens (Paraíba, Brazil). Chitosan (CS) from crab shells, as supplied by Polymar (Ceará, Brazil) with 95% deacetylation degree and viscosity of 74.03 cps was used since the high amount of active primary amine on the chitosan backbone provides excellent reactive sites for chemical modifications. The quaternary ammonium salt used was cetyl trimethyl ammonium bromide (C_16_H_33_(CH_3_)_3_N^+^Br^−^, MW: 364 g/mol), labeled CTAB, supplied by Vetec (São Paulo/Brazil) and it was used without any further purification. Analytical grade reagents (glacial acetic acid and sodium hydroxide) were purchased from Vetec and were used as received. All the samples were prepared with distilled water throughout the experiment. The microorganisms used in this study were *Escherichia coli* (ATCC 25922), *Salmonella* sp. (ATCC 14028), and *Staphylococcus aureus* (ATCC 25923) and donated by State University of Paraíba (Campina Grande, PB, Brazil).

### 2.2. Preparation of CTAB Functionalized CHITOSAN

The CTAB functionalized chitosan (CS-CTAB) was prepared according to method previously published [38]. Chitosan solution was made by dissolving the required amount of chitosan powder (3 g) into 1% aqueous acetic acid solution (300 mL). The reactants were magnetically stirred at 45 °C for 2 h, and then the CTAB (0.0662 g) was added to the reaction mixture and stirred at the same temperature for another 2 h. The CTAB amount was determined to react with 100% of the amino groups present in the chitosan and was calculated using Equation (1):

(1) $$\varphi =\;\frac{\alpha M_{S}}{M_{E}+\alpha M_{1}+(1-\alpha )M_{2}}$$

here φ is the amount of CTAB necessary to react with 1 g of chitosan; *M_S_*, *M_E_*, *M*_1_, and *M*_2_ are the molar mass of CTAB, glucose skeleton, amino group, and acetamide group, respectively, and α = 0.95 is the deacetylation degree.

When the reaction was complete, two different methods for obtaining CS-CTAB powders were employed: evaporation and precipitation. For the first method, the CS-CTAB reaction mixture was oven dried under air circulation at 50 °C for 115 h, subsequently grounded with an agate mortar, and sieved to 74 µm for use. For the precipitation method, the CS-CTAB reaction mixture was maintained at room temperature (24 h), then it was precipitated via the addition of NaOH solution 1 M (130 mL), followed by centrifugation at 3800 rpm for 5 min at room temperature. The precipitated material was collected, oven dried under air circulation at 50 °C for 96 h, and, also grounded and sieved in the conditions previously described. The CS-CTAB powders were obtained by evaporation and precipitation and coded as CS-CTABE and CS-CTABP, respectively.

### 2.3. Preparation of HDPE/Chitosan Composites

Melt processing of neat HDPE, HDPE/CS unadditived and HDPE/CS additived with CS-CTAB (HDPE/CS/CS-CTAB) was carried out in a Haake Rheomix 3000 laboratory internal mixer (Waltham, MA, USA), equipped with a mixing chamber with high-intensity rotors (roller type) operated at 60 rpm with chamber wall temperature kept at 170 °C. Batch mass was selected to fill 90% of the processing chamber volume during the last stage of the process (fully molten material). HDPE, as received, was processed for 10 min. For composites, HDPE was loaded first and after 6 min, CS, CS/CS-CTAB (dried under vacuum for 48 h at 50 °C) were added without interrupting the process and the mixing continued for another 4 min. After the melt processing, each sample was grounded, dried under vacuum for 24 h at 80 °C, and compression molded in a hydraulic press at 160 °C for 5 min to obtain samples in accordance with Standard ASTM D-638, Type 4. The prepared composites are summarized in Table 1.

### 2.4. Characterization

X-ray diffraction (XRD) was employed to assess the HDPE/chitosan composites crystallinity. A Bruker D2 Phaser X-ray diffractometer (Houston, TX, USA) with CuK*α* radiation (*λ* = 1.54 Å) operating at 40 kV and 40 mA was used to carry out the experiments at room temperature. The spectra were recorded over a 2*θ* range of 15–30° using a scan rate of 0.02°/s.

The HDPE/chitosan composites crystallinity (*X_c_*) was calculated from the XRD data by following Equation (2) using simple peak area method [47].

(2) $$X_{c}(\%)=\left(\frac{I_{c}}{I_{c}+{kI}_{a}}\right)$$

where *I_c_* is the diffraction intensity associated with the planes crystalline and *I_a_* is the intensity of the amorphous halo. *k* is constant of proportionality of HDPE in the value from 1.235 [48].

Chitosan (CS) and cetyl trimethyl ammonium bromide (CTAB) samples were characterized by XRD as powder sieved through a 200 mesh, and pure HDPE and HDPE/chitosan composites were characterized in the form of plates.

Differential scanning calorimetry (DSC) was used to study the crystallization and melting behavior of the HDPE/chitosan composites using a Shimadzu DSC (Kyoto, Japan)—60 instrument. The sample was cut into small pieces and ~5 mg of each sample was used for analysis. To remove the thermal history, the sample was heated from 30 °C to 200 °C, kept at 200 °C for 3 min and cooled to −130 °C and then reheated up to 200 °C at a heating rate 10 °C/min under argon atmosphere. The second heating scan was used to determine the crystalline melting enthalpy of the matrix.

The crystallinity (*X_c_*) of samples was determined by the following Equation (3).

(3) $$\begin{array}{l} X_{c}(\%)=\frac{{}_{\Delta H_{experimental}/W_{HDPE}}}{{}_{\Delta H_{{}_{theorical}}}}*100 \end{array}$$

where ∆*H_experimental_* is the observed heat of fusion of the sample, *W_HDPE_* is weight fraction of HDPE in the composites and ∆*H_theorical_* is the heat of fusion of 100% crystalline HDPE and was taken to be 277 J/g [49].

Thermogravimetric analysis (TG) was employed aiming to evaluate the thermal stability of the samples under argon environment and flow of 50 mL/min, using a Shimadzu 60H thermogravimetric analyzer (TG) (Kyoto, Japan). Approximately 10 mg of sample was placed in the alumina crucibles at a heating rate 10 °C/min from room temperature to 900 °C.

Tensile test was conducted in a Shimadzu Universal machine (Kyoto, Japan), Model autograph AG-X 10 KN, employing a crosshead speed 5 mm/min. The test was performed according to Standard ASTM D638 (Type IV). Six samples from each composition were tested. All the measurements were carried out at room temperature (30 *±* 2 °C).

Scanning electron microscopy (SEM) used to evaluate the effects of adding additive (CTAB-CS) on the morphology of the HDPE/CS composites was a FEI Quanta 450 instrument (Hillsboro, OR, USA). The cryofractured sample was obtained after being submerged in liquid nitrogen for approximately 15 min. A fine layer of gold was deposited over the fracture surfaces prior to test. The instrument was operated at 10–15 KV.

Antibacterial properties of the HDPE/CS8 composites with and without additive (CS-CTAB) were determined by the microbial adhesion assay according to Standard JIS Z 2801:2000 (E) [50]. 24 h culture of Gram-negative bacterium, *Escherichia coli* and *Salmonella* sp. and a Gram-positive bacterium, *Staphylococcus aureus* were selected as test microorganisms. The strains were grown on Brain-heart infusion agar (BHI) in a bacteriological incubator at 37 °C for 24 h. Then, suspensions of the microorganisms were prepared in saline solution on the 0.5 McFarlands’ scale. The HDPE/CS8 formulations were sized at 1 cm^2^, 70% ethanol sterilized and placed in a 24-well culture dish with 900 µL Mueller Hinton broth culture medium (KASVI) and 100 µL of microorganism suspension. Then, the 24-well culture dish was placed in a bacteriological incubator at 37 °C for 24 h. Then, the samples were removed from the dish, washed with saline solution at 0.9% and the microorganism was fixed by immersion glutaraldehyde at 2.5% followed by sequentially dehydration using 15, 30, 50, 70, 85 and 99.6% of ethanol. After that, the samples were incubated at 50 °C for 24 h. The evaluation of antibacterial activity was carried out using a Scanning Electronic Microscopy (SEM)-FEI Quanta 450 instrument (Hillsboro, OR, USA). The sample surface was sputter-coated with gold to prevent the occurrence of electrostatic charge during observation.

## 3. Results and Discussion

**Figure 1:**
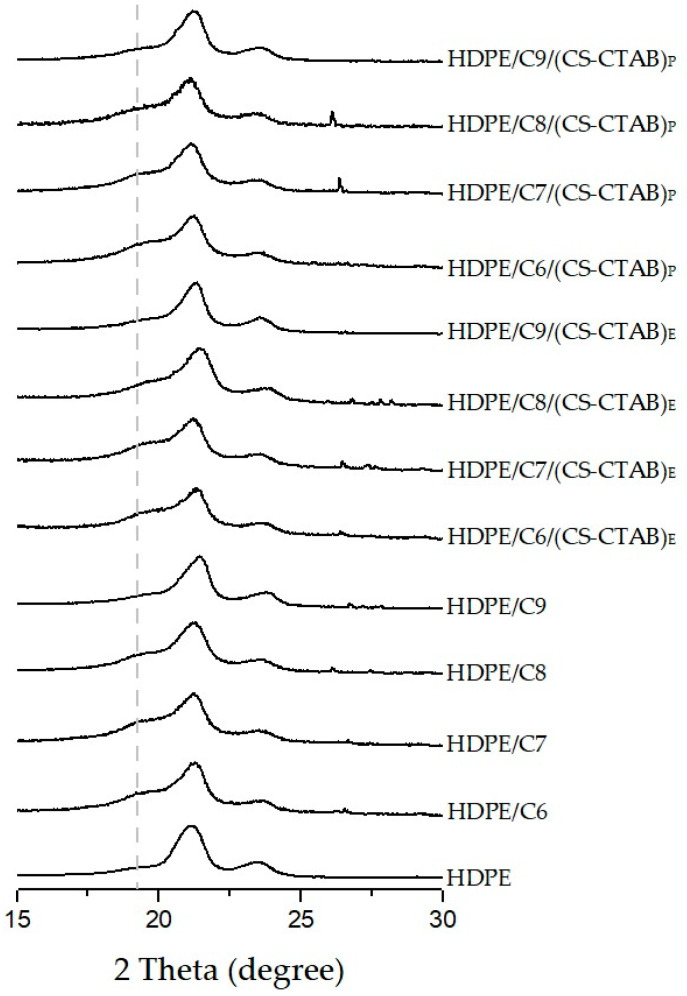
X-ray diffraction pattern of HDPE, HDPE/CS, and HDPE/CS/(CS-CTAB).

Figure 1 shows the XRD pattern for HDPE and HDPE/CS hybrids for HDPE, it observed the presence of two crystalline peaks at 21° and 23.3°, related to the (110) and (200) planes, respectively, characteristics of the orthorhombic structure of the crystal polyethylene [51,52]. For all the HDPE/CS composites, with and without the additive (CS-CTAB_E_ and CS-CTAB_P_), crystalline peaks were observed in the region of 21° and 23°, which is characteristic of the crystalline phase of the HDPE, and another peak of low intensity around 20° belonging to chitosan, as expected. By comparing all XRD patterns, a slight decrease in the intensity of the crystalline peaks of the HDPE/CS composites was observed as the chitosan content increased. This behavior may be associated with the chitosan structure to clutter the packaging of the HDPE molecules, thus reducing the crystallinity index [52]. Similar behavior was assessed by De Araujo et al. (2018) [38] when using the clay from our region impregnated with a quaternary ammonium salt. A scattering peak also appears at 2θ = 19.3°, which stands for the amorphous portion.

In comparative analysis (Table 2), it is observed in general that the *X_c_* of composites with the additive (CS-CTAB)_P_ showed a slight increase in its degree of crystallinity in relation to composites with (CS-CTAB)_E_. It is suggested that the additive prepared by the precipitation method (CS-CTAB)_P_, favored a greater rearrangement of the HDPE/CS molecules in the composition, possibly due to the lower CTAB salt content used in obtaining the functionalized chitosan (CS-CTAB)_P_. The same behavior was observed by De Araújo et al. (2018) [38]. This result was also analyzed through the DSC as shown next.

Table 3 shows the thermal properties determined by differential scanning calorimetry (DSC) under heating and cooling for pure HDPE and HDPE/CS composites. The crystallization (*Tc*) and melting (*Tm*) temperatures for pure HDPE were 115 °C and 130 °C, respectively. Regarding the different compositions, it is observed that the incorporation of different contents of chitosan and additives (CS-CTAB_E_ and CS-CTAB_P_) to HDPE had no major effect on *Tm* and *Tc*. Behavior similar to this has been reported in the literature [35].

By increasing the concentration of chitosan, the melting point of the composites tends to decrease, especially for compositions HDPE/CS8/(CS-CTAB)_E_ and _P_ (124 °C) in relation to HDPE/CS8 (129 °C). This decrease in the melting point would reduce the energy consumption during the manufacturing process, resulting in lower production costs; this indicates the feasibility of the proposed approach [53].

The degree of crystallinity (*X_c_*) for HDPE was 64% as observed also by Kahar et al. (2016) [54]. The incorporation of different contents of chitosan in the HDPE compositions greatly affected this property (Table 3). There is a decrease in the values of (*X_c_*) for HDPE/CS composites in different proportions as the chitosan content was increased. This is possibly due to chitosan causing disorder in the HDPE crystalline system [10,35]. A small difference among the values of *Tm* and *Tc* was observed in both composites with and without additive (CTAB_P_ and CTAB_E_) but the percentage crystallinity was greatly affected by the incorporation of chitosan. It shows that chitosan inhibits the close packing of the PE chains. The lower crystallinity of composites with additive was due to the chitosan content. The chitosan structure reduced the flexibility of composites which lowered the peak crystallization temperature and consequently reduced crystallinity. A similar trend has been reported in the literature [35,55,56].

In this study, TG was conducted to assess the effect of chitosan (CS) and chitosan functionalized with quaternary ammonium salt (CS-CTAB_E_ and CS-CTAB_P_) addition on the thermal properties of HDPE. Thermal stability studies are necessary in the case of materials that are used for packaging applications as the polymer may be subjected to heat processes during their preparation, processing, or conception.

**Figure 2:**
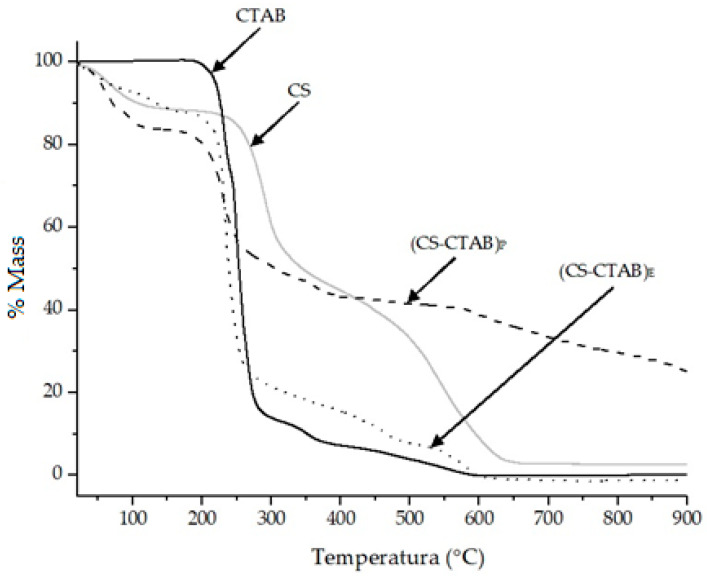
TG curves for the chitosan (CS), organic salt (CTAB), and the additives (CS-CTAB)_E_, (CS-CTAB)_P_.

Figure 2 shows the TG curves for chitosan (CS), organic salt (CTAB), and chitosan functionalized with quaternary ammonium salt by two different methods, evaporation, and precipitation (CS-CTAB_E_ and CS-CTAB_P_). Pure chitosan shows three events of mass loss. The first event occurs at an initial decomposition temperature (*Ti*) of 52 °C and refers to the water loss associated with the amino groups in the polysaccharide structure. The second event takes place at around 263 °C and is altered by the decomposition of organic matter that begins with the breakdown of glycosidic bonds accompanied by the decomposition of the acetylated and deacetylated units of the polymer. The third event occurs in a *Ti* of approximately 498 °C, resulting from the decomposition of the polymer residues [57,58].

For chitosan functionalized with quaternary ammonium salt (CS-CTAB) obtained by the evaporation and precipitation methods (Figure 2), three mass loss events are observed. The first loss of mass occurs in a *Ti* of 42 °C, belonging to water and acetic acid present in both samples. The second event takes place in a *Ti* at 225 °C and is associated with organic salt (CTAB) [59], present in the chemical functionalization of chitosan. For chitosan functionalized with quaternary ammonium and prepared by the evaporation method (CS-CTAB)_E,_ there is a loss of mass related to the second event, around 60%. Such loss may be associated with the excess of organic salt (CTAB) present in the sample, from the evaporation method itself. On the other hand, for chitosan functionalized with quaternary ammonium and prepared by precipitation (CS-CTAB)_P_, a loss of mass related to the second event of only 29% is observed, much lower than the percentage presented by the sample obtained by the evaporation method. The third event occurs in a *Ti* of 425 °C, resulting from the decomposition of organic salt and polymer residues [59,60]. There is a residual mass of approximately 25% for the (CS-CTAB)_P_ sample. The (CS-CTAB)_E_ sample is completely decomposed. This is possibly due to the NaOH residues retained in the sample after the precipitation process. These samples (CS-CTAB)_E_ and (CS-CTAB)_P_ were used as additives in the HDPE/CS composites. Therefore, TG was conducted to assess the effect of chitosan (CS) and samples (CS-CTAB_E_ and CS-CTAB_P_) as additives on the thermal properties of HDPE-based composites. Thermal stability studies are necessary in the case of materials that are used for packaging applications as the polymer may be subjected to heat processes during their preparation, processing, or conception [38].

Table 4 shows the degradation temperatures step of HDPE and HDPE/CS composites, with and without additive.

From Table 4, only one mass loss event is observed whose decomposition temperature stage (*Ti*–*Tf*) is in the range of 463–489 °C, corresponding to the decomposition of the pure HDPE [1,35]. It is possible to observe also that the CS and all other compositions exhibited the same decomposition profile, presenting three stages of mass loss. Such losses are characteristic of the components present in the mixture HDPE/CS as considered individually.

The first stage below 100 °C was mainly attributed to the loss of water absorbed from the materials. The second stage, ranging from 263 °C to 314 °C, was due to the thermal degradation of chitosan. In this stage, degradation of chitosan took place, which involved dehydration, deacetylation, and chain scission reactions. The third stage (406–491 °C) was attributed to the decomposition of the HDPE matrix. These data are in accordance with the reported in the literature [61].

It is clear from the Table 3 data that the thermal stability of HDPE/CS and HDPE/CS/(CS-CTAB) composites are similar to those of HDPE. Chitosan and chitosan’s functionalized (CS-CTAB)_E, P_ had no significant effect on the overall characteristics of each weight loss stage of the HDPE-based composites, regardless of the method (evaporation or precipitation) or the weight ratio used. Special attention is given to the composite HDPE/CS8/(CS-CTAB)_E_ where a mass loss event in the range of 469–490 °C is observed. This event may be associated with the content of organic salt and chitosan present in the sample.

The degradation of HDPE was not significantly affected when blended with chitosan and chitosan functionalized with quaternary ammonium salt (CS-CTAB). The composites HDPE/CS and HDPE/CS/(CS-CTAB) prepared by melting may perhaps avoid degradation and/or a drastic loss in the antibacterial properties of the final material prepared by melt processing. This behavior is according to the literature [38,62].

Still, according to Table 3, it can be seen that the decomposition temperature at 20% of mass loss (*T20*), presented greater thermal stability for all compositions based on HDPE/CS9 and HDPE/CS8 with and without (CS-CTAB)_E, P_. Remarkable thermal stability was observed for the compositions HDPE/CS9 and HDPE/CS8 containing (CS-CTAB)_P_, 332 and 312 °C, respectively. Such results were expected, since these compositions had lower amounts of chitosan and organic salt in the mixture when compared to the composites HDPE/CS7 and HDPE/CS6. This indicates that increasing the chitosan concentration tends to slightly improve the composite thermal degradation [53].

The properties of the tensile strength, Young’s modulus, and elongation at break of HDPE, HDPE/CS, and HDPE/CS/(CS-CTAB) composites are presented in Table 5.

The HDPE/CS8 composite showed a slight increase in tensile strength when compared to that of pure HDPE. However, for the other HDPE/CS9, HDPE/CS7 and HDPE/CS6 compositions, the addition of chitosan did not significantly affect this property. In a comparative analysis, it is verified in general that the tensile strength of HDPE was not affected by the addition of chitosan and chitosan functionalized (CS-CTAB), regardless of the additive type and its quantity.

It is observed that Young’s modulus increased with the incorporation of CS and (CS-CTAB)_E, P_ in HDPE compositions with respect to the pure HDPE. Thus, it is possible to see an increase in this property as the chitosan content in the mixtures increased (Table 4), but it was not affected by (CS-CTAB) preparation method (evaporation or precipitation). It is suggested that chitosan has a Young’s modulus value greater than that of pure HDPE. According to Vasile et al. (2013) [10], the mechanical properties of the LDPE and chitosan blends decreased except for the Young’s modulus, which increased with the addition of chitosan because this biopolymer is more rigid than PE. Behavior similar was also observed by De Araujo et al. (2018) [38]. Sunilkumar et al. (2012) [13] in their studies reported that this is because chitosan is an immiscible component, has low ductility, and is a rather brittle material; thus, its addition made the blend more rigid than pure LDPE film.

From Table 5, a significant reduction in the elongation at break values for composites in all proportions without and with additives when compared to pure HDPE is observed. Such behavior was expected, since chitosan, despite being a strong material, presents ductility lower than that of HDPE. Among the compositions analyzed, the composites HDPE/CS9 and HDPE/CS8 showed the best values of elongation at break, with and without additives. In particular, the HDPE/CS9/(CS-CTAB)_P_ sample obtained the highest elongation at break (12.10%—Table 5) compared to all other compositions. Such results corroborate the TG data, previously discussed. This elongation property can be improved with the incorporation of compatibilizers such as vinyl triethoxysilane [35] and maleic anhydride [39], for example.

According to Agrawal et al. (2008) [63], and Ferreira et al. (2011) [64], an increase in the elongation at break indicates an increase in the toughness of the material. As observed, functionalized chitosan (CS-CTAB)_E_ and _P_, showed a positive effect on the elongation at break for the composition HDPE/CS9 while for the other compositions this reduction in the toughness can favor greater biodegradation of the materials with the incorporation of the additive (CS-CTAB)_P_ and _E_.

According to Kusumastuti et al. (2020) [53], with the increase of the chitosan content, both tensile strength and Young’s modulus increased; however, the elongation decreased. Behavior similar has been observed in our study. According to Sloan et al. (1986) [65], the Young’s modulus or elastic modulus is greatly influenced by the uniform particle’s dispersion into the matrix. Reesha et al. (2015) [11] suggested that the low tensile strength results from an uneven dispersion of chitosan in the LDPE matrix. Thus, the homogeneity or even particle distribution of the blended polymer is a key to increasing both tensile strength and Young’s modulus [53].

**Scheme 1:**
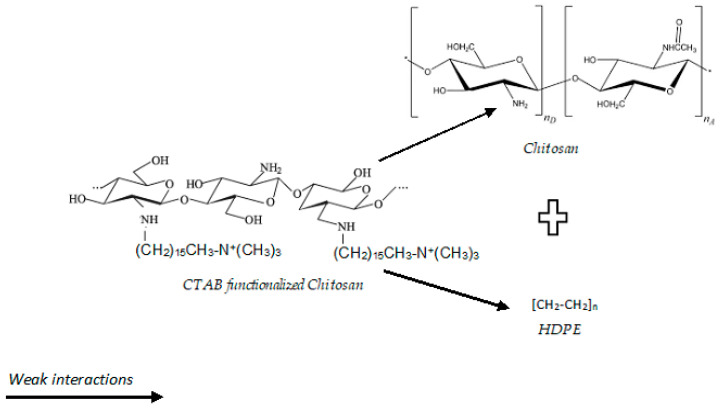
Schematic representation of the possible interaction of the additive (CS-CTAB) with the HDPE/Chitosan composite.

A possible interaction of additive (CS-CTAB) with the HDPE/chitosan composite is represented according to Scheme 1 below.

**Figure 3:**
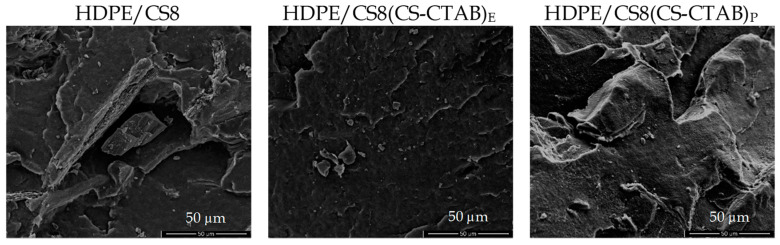
SEM images of HDPE/CS8, HDPE/CS8/(CS-CTAB)_P_ and HDPE/CS8/(CS-CTAB)_E_.

In view of the results obtained in the tensile tests and in order to evaluate the effect of the additive (CS-CTAB) in the HDPE/CS composites, scanning electron micrograph (SEM) analyses were performed whose micrographs are shown in Figure 3.

In the micrograph of the HDPE/CS8 composite (Figure 3), it is possible to perceive a rough surface with the presence of voids as well as small particles of chitosan dispersed in the HDPE matrix. Such behavior indicates a possible weak interfacial adhesion between the polymers [66].

For the sample, HDPE/CS8 (CS-CTAB)_E_ (Figure 3), a smoother, uniform, and homogeneous surface is verified, but it contains clusters of crystals (CS-CTAB)_E_ of varying sizes dispersed along the HDPE matrix. The presence of these crystals is possibly due to the evaporation method used in the preparation of this additive (CS-CTAB)_E_, which favors a greater amount of organic salt in the functionalization of chitosan. This salt content may also be responsible for the presence of agglomerates throughout the matrix. However, an improvement in interfacial tension was observed for this mixture as reported in Table 4. The composition HDPE/CS8(CS-CTAB)_P_ (Figure 3) also presented a surface similar to the sample HDPE/CS8(CS-CTAB)_E_ with the difference that in this micrograph there is the presence of few and small particles (CS-CTAB)_P_ distributed in the matrix polymeric. This characterizes a good interfacial adhesion of the mixture, considering that the additive (CS-CTAB)_P_ acted positively in the compatibilization of the system. These results corroborate with the mechanical properties data presented in Table 4.

In view of this, it is suggested that the chitosan functionalized with quaternary ammonium salt played an important role as a sustainable additive in decreasing the domain sizes of the dispersed phase and reducing the interfacial tension, thus, contributing to the better interfacial adhesion between HDPE and chitosan used in this study. The incorporation of this additive, in addition to improving some properties as reported above, can promote greater biodegradation of materials applied to the packaging sector.

**Figure 4:**
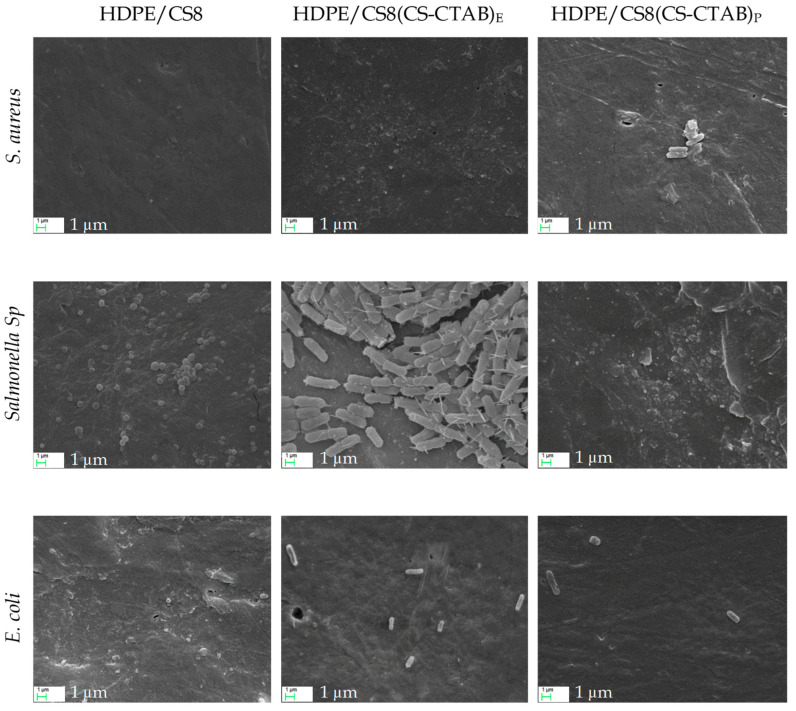
SEM observation of adhered *S. aureus*, *Salmonella* sp., and *E. coli* after incubation for 24 h onto HDPE, HDPE/CS8/(CS-CTAB)_P,_ and HDPE/CS8/(CS-CTAB)_E_ composites surfaces.

In order to evaluate the antimicrobial property of the additive (CS-CTAB) in the HDPE/CS8 composites intended for food packaging, these were subjected to microbial adhesion tests against three strains: one Gram-positive (*Staphylococcus aureus*), and two Gram-negative (*Salmonella* sp. and *Escherichia coli*). Then, these formulations were analyzed by scanning electron microscopy (SEM), whose micrographs are shown in Figure 4.

The tests showed that the growth of the two bacteria (*S. aureus* and *E. coli*) was inhibited in the presence of HDPE formulations containing chitosan. This fact proves that chitosan does not lose its functionality even if the mixtures are processed in the molten state at a temperature of 170 °C, below its decomposition temperature, since the thermal behavior studies presented above and by other authors have shown that the main process chitosan decomposition occurs in the 270–337 °C range, which is attributed to the chitosan depolymerization reaction [67]. Vasile et al. (2013) [10] also analyzed this behavior in their studies on low-density polyethylene and chitosan composites.

Chitosan has been investigated as an antimicrobial material against a wide range of target organisms like algae, bacteria, yeasts, and fungi in experiments involving in vivo and in vitro interactions with chitosan in different forms (solutions, films, and composites) [68]. Generally, in these studies, the chitosan is considered to be a bactericidal (kills the live bacteria or some fraction therein) or bacteriostatic (hinders the growth of bacteria but does not imply whether or not bacteria are killed), often with no distinction between activities. Recent data in the literature has the tendency to characterize chitosan as bacteriostatic rather than bactericidal [69], although the exact mechanism is not fully understood and several other factors may contribute to the antibacterial action [70,71]. Three models have been proposed, the most acceptable being the interaction between positively charged chitosan molecules and negatively charged microbial cell membranes. In this model, the interaction is mediated by the electrostatic forces between the protonated NH^+3^ groups and the negative residues, presumably by competing with Ca^2+^ for electronegative sites on the membrane surface [22,43,68,72].

The bacterial effectiveness of gram-positive or gram-negative bacteria is, however, somewhat controversial. Some authors have stated that chitosan generally showed stronger effects for gram-positive bacteria than for gram-negative bacteria [73,74,75,76]. Conversely, it has been demonstrated that hydrophilicity in gram-negative bacteria is significantly higher than in gram-positive bacteria, making them most sensitive to chitosan [77]. These findings are confirmed by several in vitro experiments in which gram-negative bacteria appear to be very sensitive to chitosan, exhibiting increased morphological changes on treatment when compared to gram-positives [78,79,80,81,82]. The charge density on the cell surface is a determinant factor to establish the amount of adsorbed chitosan. More adsorbed chitosan would evidently result in greater changes in the structure and in the permeability of the cell membrane. This would suggest that the antibacterial mode of action is dependent upon the host microorganism [71,83]. A possible mechanism of antimicrobial action is under study by our group, and we hope to make this contribution soon.

On the other hand, the incorporation of the additive (CS-CTAB)_P_ in the HDPE/CS8 formulations led to total inhibition of the growth of *Salmonella* bacteria and partial inhibition against *S. aureus* and *E. coli* strains. Among them, the additive acted positively only against the proliferation of *Salmonella* bacteria, however, in the other formulations its antimicrobial action was quite discreet. When adding the other additive (CS-CTAB)_E_ in the HDPE/CS8 formulations, there is a positive effect against the *S. aureus* strain, demonstrating efficiency in its antimicrobial action, while for the other formulations against the *E. coli* and *Salmonella* strains, no antimicrobial effect was observed, only a partial inhibition against the *E. coli* strain, and no antimicrobial effect against *Salmonella* as seen in the micrograph (Figure 3), characterized by the presence of a large number of rods along the surface.

These results showed that quaternization does not always enhance the antibacterial activity of CS and that the effect of pH on the antibacterial activity of quaternary ammonium CS is uncertain. The discrepancies among different reports on the antibacterial activity of CS and its quaternary derivatives are most likely caused by various intrinsic and extrinsic factors that are related to the CS itself (e.g., type, MW, DD, viscosity solvent, and concentration) and the environmental conditions (e.g., test strain, its physiological state, and the bacterial culture medium, pH, temperature, ionic strength, metal ions, and organic matter), respectively [83].

Quaternized chitosan was studied widely as an antibacterial agent [43,46,71,84,85] but the exact mechanism of antibacterial activity of quaternary ammonium chitosan was still not confirmed. The effect of positive charge was advocated further [85]. The cell envelope of Gram-positive bacterium (*S. aureus*) was fully composed of lipoteichoic acids [86], which provided a molecular linkage with quaternized chitosan and resulted in the damage of membrane functions while the outer membrane of Gram-negative bacterium (*E. coli*) contained lipopolysaccharide (LPS) and the outer LPS layer was a potential barrier against foreign molecules. It was possible that the disparities in bacterial membrane functions would generate the differences in antibacterial effect for *S. aureus* and *E. coli*. All in all, it is generally considered that the antibacterial effects against *S. aureus* and *E. coli* were due to the mutual attraction between the positive charge of quaternized chitosan and the negative charge of the microbial cell membrane surface, resulting in the damage of the cell membrane. The broken cell membrane led to the leakage of intracellular constituents or the entrance of macromolecules, and the bacteria would hardly breed [71,87].

In view of the above, the positive effect of the antimicrobial activity of the additives prepared by precipitation and evaporation against the strains *Salmonella* sp. and *S. aureus*, respectively, used in the HDPE/CS8 composites was evidenced. On the other hand, remarkable antimicrobial activity was observed for the HDPE/CS8 composites without additive against the two strains *S. aureus* and *E. coli*. Bearing in mind that this work has the aim of production of food packaging with antimicrobial activity, and in view of the results obtained, it can be considered that different HDPE/CS8 formulations exhibited satisfactory antimicrobial activity for a given strain in the study.

## 4. Conclusions

Different HDPE/Chitosan composites containing up to 40% of CS and an additive based on chitosan functionalized with quaternary ammonium salt were investigated in this work. The incorporation of this additive in the different formulations of HDPE/CS played a synergistic role together with chitosan, obtaining materials with satisfactory thermal and mechanical properties making them suitable candidates for food packaging. This additive (CS-CTAB) also promoted a positive effect on antimicrobial activity when incorporated into the composites. It can see also that the HDPE/CS composites without the additive exhibited satisfactory antimicrobial activity against the two strains *S. aureus* and *E. coli*, as found in contaminated food. The newly obtained materials showed good inhibition activity against gram-positive and gram-negative bacteria in the study. Finally, it can be concluded that HDPE/CS composites prepared in our study can not only passively protect food against environmental factors but can increase the shelf life of food products.

## Figures and Tables

**Table 1:** Compositions of the prepared samples.

Code	Composition	HDPE (g)	CS (g)	CS-CTAB (g)
HDPE	100	50	0	0
HDPE/CS9	90/10	45	5	0
HDPE/CS8	80/20	40	10	0
HDPE/CS7	70/30	35	15	0
HDPE/CS6	60/40	30	20	0
HDPE/CS9/(CS-CTAB)_E_	90/10/0.1	45	5	0.05
HDPE/CS8/(CS-CTAB)_E_	80/20/0.2	40	10	0.10
HDPE/CS7/(CS-CTAB)_E_	70/30/0.3	35	15	0.15
HDPE/CS6/(CS-CTAB)_E_	60/40/0.4	30	20	0.20
HDPE/CS9/(CS-CTAB)_P_	90/10/0.1	45	5	0.05
HDPE/CS8/(CS-CTAB)_P_	80/20/0.2	40	10	0.10
HDPE/CS7/(CS-CTAB)_P_	70/30/0.3	35	15	0.15
HDPE/CS6/(CS-CTAB)_P_	60/40/0.4	30	20	0.20

**Table 2:** Crystallinity (*X_c_*) of HDPE and HDPE/CS composites as evaluated using Equation (1).

Sample	*X_c_* (%)
HDPE	54
HDPE/CS9	45
HDPE/CS9/(CS-CTAB)_E_	45
HDPE/CS9/(CS-CTAB)_P_	50
HDPE/CS8	43
HDPE/CS8/(CS-CTAB)_E_	41
HDPE/CS8/(CS-CTAB)_P_	39
HDPE/CS7	38
HDPE/CS7/(CS-CTAB)_E_	34
HDPE/CS7/(CS-CTAB)_P_	40
HDPE/CS6	39
HDPE/CS6/(CS-CTAB)_E_	32
HDPE/CS6/(CS-CTAB)_P_	36

**Table 3:** DSC of HDPE and HDPE/CS composites.

Sample	*Tc* (°C)	∆*H_c_* (J/g)	*Tm* (°C)	∆*H_m_* (J/g)	*X_c_* (%)
HDPE	115	155	130	184	64
HDPE/CS9	115	110	130	141	53
HDPE/CS9/(CS-CTAB)_E_	111	69	125	97	37
HDPE/CS9/(CS-CTAB)_P_	115	63	129	96	36
HDPE/CS8	114	40	129	99	42
HDPE/CS8/(CS-CTAB)_E_	110	51	124	82	35
HDPE/CS8/(CS-CTAB)_P_	110	53	124	84	36
HDPE/CS7	113	45	128	56	27
HDPE/CS7/(CS-CTAB)_E_	114	42	128	61	30
HDPE/CS7/(CS-CTAB)_P_	116	57	129	77	37
HDPE/CS6	114	50	127	63	36
HDPE/CS6/(CS-CTAB)_E_	115	41	128	53	30
HDPE/CS6/(CS-CTAB)_P_	113	44	128	52	30

**Table 4:** Degradation temperature events of the HDPE, CS, HDPE/CS and HDPE/CS/(CS-CTAB).

Sample		*Ti*–*Tf* (°C)		
	I	II	III	*T20* (°C)
HDPE			463–489	460
CS	62–98	263–309	496–592	266
HDPE/CS9	40–78	263–311	459–490	440
HDPE/CS9/(CS-CTAB)_E_	37–76	270–312	460–491	321
HDPE/CS9/(CS-CTAB)_P_	36–73	271–310	456–488	332
HDPE/CS8	38–77	268–312	458–489	302
HDPE/CS8/(CS-CTAB)_E_	37–76	269–312	469–490	302
HDPE/CS8/(CS-CTAB)_P_	30–81	266–310	456–487	312
HDPE/CS7	36–78	265–310	451–486	302
HDPE/CS7/(CS-CTAB)_E_	38–77	269–311	454–486	293
HDPE/CS7/(CS-CTAB)_P_	39–78	267–312	456–488	291
HDPE/CS6	50–93	267–309	406–488	290
HDPE/CS6/(CS-CTAB)_E_	42–82	267–314	456–487	293
HDPE/CS6/(CS-CTAB)_P_	37–82	268–311	455–488	289

*Ti*—onset temperature and *Tf*—end temperature.

**Table 5:** Tensile properties of HDPE, HDPE/CS and HDPE/CS/(CS-CTAB) compositions.

Sample	Tensile Strength (MPa)	Elastic Modulus (MPa)	Elongation at Break (%)
HDPE	16.38 ± 2.11	400.72 ± 52.49	67.14 ± 12.33
HDPE/CS9	16.70 ± 0.58	443.50 ± 25.59	15.99 ± 0.65
HDPE/CS9/(CS-CTAB)_E_	14.49 ± 0.31	399.28 ± 11.43	12.79 ± 0.09
HDPE/CS9/(CS-CTAB)_P_	16.61 ± 0.39	421.18 ± 12.61	12.10 ± 0.22
HDPE/CS8	18.35 ± 0.98	538.28 ± 21.47	12.01 ± 3.92
HDPE/CS8/(CS-CTAB)_E_	16.94 ± 0.39	435.20 ± 16.41	9.63 ± 1.11
HDPE/CS8/(CS-CTAB)_P_	15.15 ± 0.63	467.57 ± 13.29	9.18 ± 1.31
HDPE/CS7	17.26 ± 0.45	537.47 ± 16.41	11.88 ± 0.88
HDPE/CS7/(CS-CTAB)_E_	17.99 ± 0.67	538.97 ± 19.37	6.75 ± 1.20
HDPE/CS7/(CS-CTAB)_P_	17.08 ± 0.71	571.40 ± 22.68	7.21 ± 0.49
HDPE/CS6	17.52 ± 0.60	597.84 ± 22.78	9.17 ± 1.35
HDPE/CS6/(CS-CTAB)_E_	15.41 ± 0.57	576.45 ± 43.41	5.93 ± 0.53
HDPE/CS6/(CS-CTAB)_P_	14.34 ± 0.54	563.34 ± 18.61	5.61 ± 0.46
